# Metabolic Brain Changes Can Predict the Underlying Pathology in Neurodegenerative Brain Disorders: A Case Report of Sporadic Creutzfeldt–Jakob Disease with Concomitant Parkinson’s Disease

**DOI:** 10.3390/ijms241713081

**Published:** 2023-08-23

**Authors:** Tomaž Rus, Jernej Mlakar, Jan Jamšek, Maja Trošt

**Affiliations:** 1 Department of Neurology, University Medical Center Ljubljana, Zaloška cesta 2a, 1000 Ljubljana, Slovenia; maja.trost@kclj.si; 2 Institute of Pathology, Medical Faculty, University of Ljubljana, Korytkova ulica 2, 1000 Ljubljana, Slovenia; jernej.mlakar@mf.uni-lj.si; 3 Department of Nuclear Medicine, University Medical Center Ljubljana, Zaloška cesta 7, 1000 Ljubljana, Slovenia; jan.jamsek@kclj.si; 4 Medical Faculty, University of Ljubljana, Vrazov trg 2, 1000 Ljubljana, Slovenia

**Keywords:** Creutzfeldt–Jakob disease, Parkinson’s disease, multiple co-proteinopathy, FDG PET, brain networks analysis

## Abstract

The co-occurrence of multiple proteinopathies is being increasingly recognized in neurodegenerative disorders and poses a challenge in differential diagnosis and patient selection for clinical trials. Changes in brain metabolism captured by positron emission tomography (PET) with ^18^ F-fluorodeoxyglucose (FDG) allow us to differentiate between different neurodegenerative disorders either by visual exploration or by studying disease-specific metabolic networks in individual patients. However, the impact of multiple proteinopathies on brain metabolism and metabolic networks remains unknown due to the absence of pathological studies. In this case study, we present a 67-year-old patient with rapidly progressing dementia clinically diagnosed with probable sporadic Creutzfeldt–Jakob disease (sCJD). However, in addition to the expected pronounced cortical and subcortical hypometabolism characteristic of sCJD, the brain FDG PET revealed an intriguing finding of unexpected relative hypermetabolism in the bilateral putamina, raising suspicions of coexisting Parkinson’s disease (PD). Additional investigation of disease-specific metabolic brain networks revealed elevated expression of both CJD-related pattern (CJDRP) and PD-related pattern (PDRP) networks. The patient eventually developed akinetic mutism and passed away seven weeks after symptom onset. Neuropathological examination confirmed neuropathological changes consistent with sCJD and the presence of Lewy bodies confirming PD pathology. Additionally, hyperphosphorylated tau and TDP-43 pathology were observed, a combination of four proteinopathies that had not been previously reported. Overall, this case provides valuable insights into the complex interplay of neurodegenerative pathologies and their impact on metabolic brain changes, emphasizing the role of metabolic brain imaging in evaluating potential presence of multiple proteinopathies.

## 1. Background

The co-occurrence of multiple proteinopathies is increasingly recognized in neurodegenerative disorders [1,2]. In addition to the classical copathology seen in mixed dementia involving the combination of Alzheimer’s disease (AD) pathology and vascular dementia, as well as dementia with Lewy bodies (DLB), where alpha-synucleinopathy is combined with amyloid-β (Aβ) pathology, combinations of coinciding proteinopathies have been frequently found in both symptomatic and asymptomatic elderly individuals. The proportion of mixed proteinopathies varies between 20% and 70% in various neurodegenerative disorders [2]. In fact, due to the presence of mixed pathology in individual patients on one side, and the diverse clinical presentations associated with individual proteinopathies on the other, our understanding of neurodegenerative disorders has shifted from considering them as individual entities to an ensemble of neurodegeneration [3].

In sporadic Creutzfeldt–Jakob disease (sCJD), a rare rapidly progressing proteinopathy characterized by the accumulation of misfolded prion protein (PrPsc), the co-occurrence of Aβ and tau pathology is not uncommon [4]. Indeed, up to a third of patients may have significant Aβ pathology and another third incipient Aβ or tau pathology (primary age-related tauopathy; PART) [4,5]. Other co-proteinopathies have been reported but are rare; there are reports of coexistent sCJD and alpha-synucleinopathy [1,6,7], as well as sCJD with TAR DNA-binding protein 43 (TDP-43) proteinopathy and argyrophilic grain disease in individual cases [6,8].

Various biomarkers are nowadays available for neurodegenerative syndromes, including disease-specific metabolic brain changes that can be investigated using brain ^18^ F-fluorodeoxyglucose (FDG) positron emission tomography (PET) [9]. Alongside traditional visual exploration and univariate statistical methods like statistical parametric mapping (SPM) [10], which independently explore brain metabolism voxel by voxel, advanced multivariate analytical approaches like scaled-subprofile model/principal component analysis (SSM/PCA) have enabled the identification of disease-specific metabolic brain networks [11]. Several research groups worldwide have identified and validated robust metabolic brain networks specific for disorders like AD, Parkinson’s disease (PD), the behavioral variant of frontotemporal dementia (bvFTD), sCJD, and others [11,12,13,14]. The extent of specific network expression observed in these disorders has been shown to correlate with the severity of its clinical impairment [11]. By measuring the expression of these specific networks in individual patients, we can improve the accuracy of diagnosis in neurodegenerative disorders [15]. However, the impact of multiple proteinopathies on the metabolic brain networks remains unknown due to the absence of pathological studies.

In this case study, we present a patient with a rapidly progressing dementia, initially diagnosed with probable sCJD according to current diagnostic criteria [16]. However, in addition to the metabolic brain changes characteristic of sCJD, the FDG PET brain scan revealed metabolic changes characteristic for PD in both visual assessment and network analysis. Subsequently, upon autopsy, sCJD was confirmed together with PD and two additional incipient proteinopathies. 

## 2. Case Report

A 67-year-old retired truck driver was admitted to the neurology department due to rapidly progressing short-term memory loss that had started ten days before admission. During this period, he noticed difficulties playing the accordion, which he used to play regularly. Upon admission, he displayed full orientation, but diminished attention and severe short-term memory deficits, as well as acalculia. He scored 17/30 on the mini-mental state examination (MMSE). Within a few days, he began experiencing spatial and temporal disorientation, along with mild limb ataxia initially on the left side, which later extended to the right too. Disinhibition, perseverations, confabulations, and frontal release signs also manifested. In addition to attention deficits, a neuropsychological examination performed 15 days after disease onset found significant executive dysfunction, difficulties in working memory, as well as learning, recall, and recognition impairment, while visuospatial functions remained preserved. Seventeen days after disease onset, myoclonus emerged and bilateral limb and gait ataxia worsened.

Blood tests conducted upon admission yielded unremarkable results, including thyroid hormone levels, autoimmune tests, tumor marker tests, and basic cerebrospinal fluid (CSF) examination. Microbiological assessment of the CSF ruled out syphilis and Lyme disease. Repeated molecular tests on the CSF were negative for common neurotropic viruses such as herpes simplex 1 and 2, varicella-zoster virus, cytomegalovirus, Epstein–Barr virus, human herpesvirus 6, enteroviruses, and polyoma virus JC. HIV infection was also excluded. Additionally, both blood and CSF samples were tested for paraneoplastic and anti-neuronal-surface-antigen (ANSA) antibodies, and the results were unremarkable.

Brain MRI conducted on the day after admission revealed cortical hyperintensities in the right frontal cortex and right cingulum, as well as to a lesser extent in the left frontal cingulum, on FLAIR and DWI sequences. An EEG performed upon admission displayed diffuse slowing over the right hemisphere, with periodic activity that in two weeks progressed into periodic sharp wave activity. CSF biomarkers showed normal Aβ and phosphorylated tau values, while total tau was significantly elevated (4191 ng/L; cut-off value <400 ng/L).

After excluding reversible causes of rapid cognitive decline, the patient was diagnosed with probable sCJD based on current clinical criteria [16].

**Figure 1:**
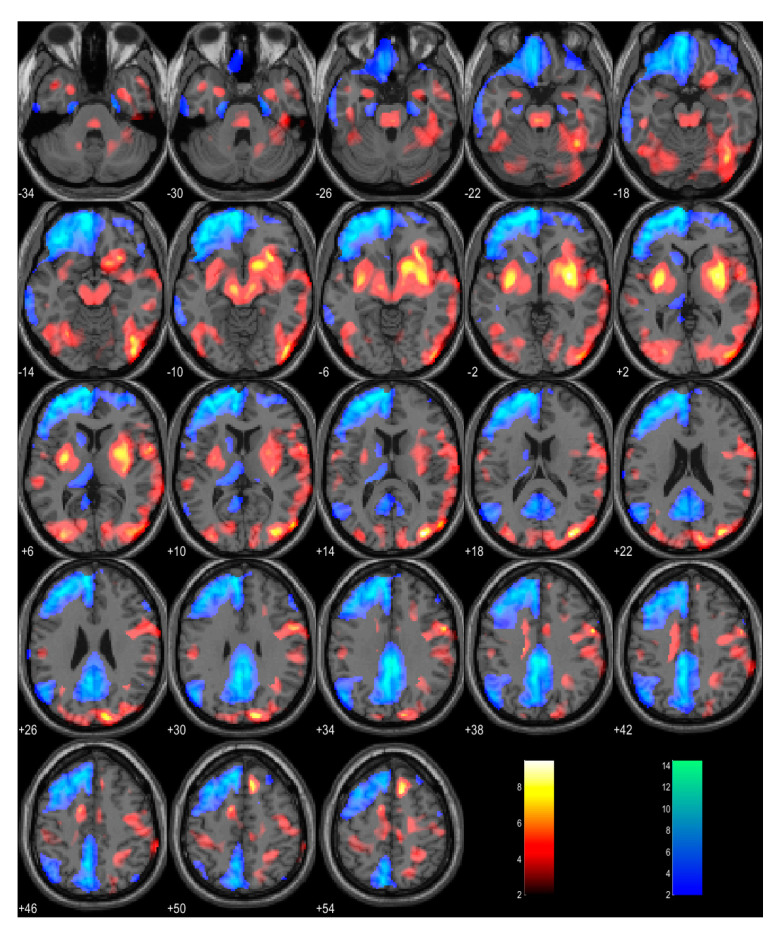
Single-subject statistical parametric mapping (SPM) analysis of patients FDG PET scan in comparison to 20 age-matched healthy subjects. Pronounced asymmetrical hypometabolism (color-coded blue) is evident in the right frontal and parietal cortex, right caudate and thalamus, bilateral precuneus and posterior cingulum and relative hypermetabolism (color-coded red) in both putamina. Clusters larger than 100 voxels, |z| > 2 are presented.

A brain FDG PET scan was performed 17 days after disease onset. Visual inspection and univariate statistical analysis using single-subject SPM showed pronounced asymmetrical hypometabolism in the frontal and parietal cortices, more pronounced on the right, as well as in the right caudate and thalamus. Bilateral hypometabolism was observed in the precuneus and posterior cingulum. Moreover, there was a notable relative hypermetabolism observed in both putamina (Figure 1), which we usually observe in PD patients.

Additionally, we performed metabolic network analysis of the patient’s FDG PET scan as described by Spetsieris et al. [17]. Alongside exploring the expression of the CJD-related pattern (CJDRP) [12], we also investigated expression of PD-related pattern (PDRP) [18] due to hypermetabolism in basal ganglia found on visual inspection.

**Figure 2:**
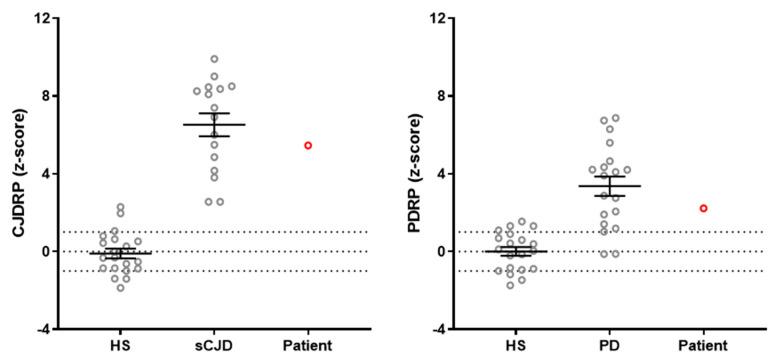
Expressions of CJDRP and PDRP networks in the patient under consideration (red circles) in comparison with the corresponding healthy subjects (HS; 20 subjects, 12 females, age 66.7 ± 5.7, MMSE 28.6 ± 1.2), sCJD (16 subjects, 7 females, age 68.1 ± 10.7; MMSE 15.0 ± 7.8) and PD patients (20 subjects, 4 females, UPDRS-III scale 30.4 ± 10.5). The clinical and demographic characteristics of the subjects are presented in [12,18].

Expression levels of both CJDRP and PDRP brain networks were significantly elevated compared to those of healthy subjects (HS). The standardized value for CJDRP expression was 5.5 standard deviations (SD) above HS (i.e., z = 5.5), falling within the range observed in CJD patients (z = 6.5 ± 2.4) [12]. Similarly, the expression of PDRP was elevated (z = 2.2) compared to HS, falling within the range observed in PD patients (z = 3.4 ± 2.2) (see Figure 2).

**Figure 3:**
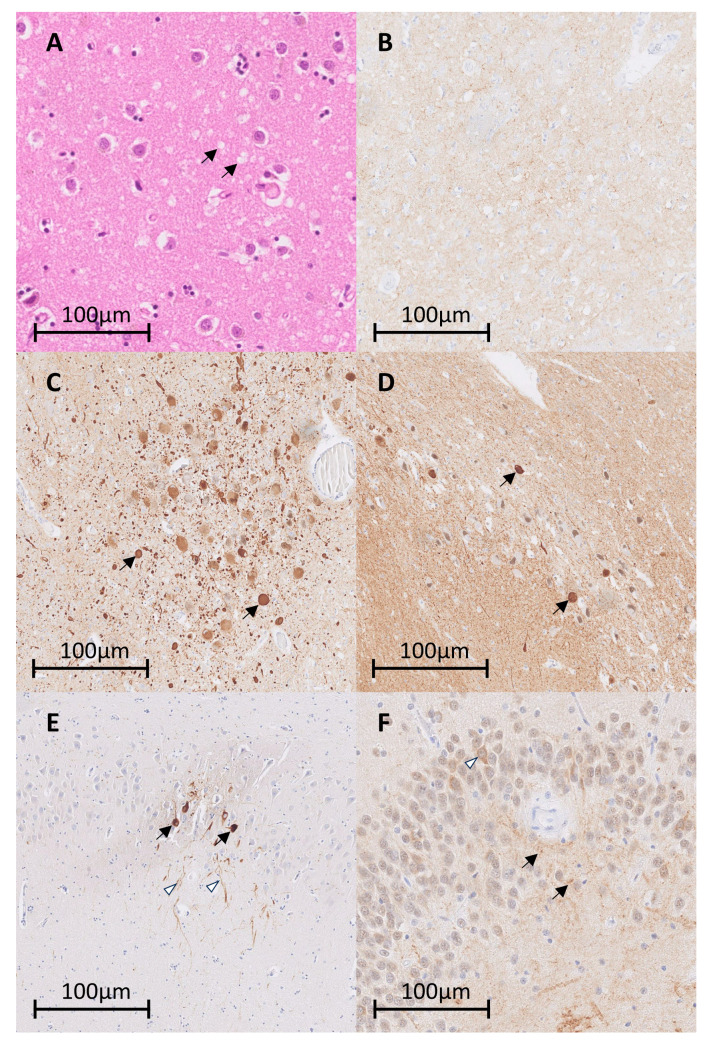
(**A**) Spongiform degeneration observed in the striatum (hematoxylin-eosin staining; empty vacuoles are marked with arrows). (**B**) Positive immunohistochemical reaction confirming the presence of the pathological prion protein (PrPsc) in the striatum. (**C**,**D**) Lewy bodies, positively stained for alpha-synuclein (marked with arrows), consistent with Parkinson’s disease, were observed in the locus coeruleus (**C**) and the substantia nigra (**D**). (**E**) Hyperphosphorylated protein tau within neurofibrillary tangles (arrows) and dystrophic neurites (empty arrowhead) in the hippocampus. (**F**) Focal accumulation of TDP-43 in the cytoplasm of individual neurons (empty arrowhead) and neuronal processes (arrows) in the dentate gyrus of the hippocampus.

The patient progressed to akinetic mutism and was offered palliative care. Seven weeks after the initial onset of symptoms, he passed away. A neuropathological examination was conducted, revealing spongiform degeneration of the cortical and subcortical gray matter throughout the cerebrum and cerebellum. These changes were most prominent in the occipital areas, basal ganglia, and hypothalamus (Figure 3A). Immunohistochemical reactions using two different monoclonal antibodies (12F10 and 3F4) confirmed the presence of the pathological form of the prion protein (PrPsc) consistent with sCJD (Figure 3B). Additionally, Lewy bodies were observed in the substantia nigra and locus ceruleus, with positive immunohistochemical reaction for alpha-synuclein in the inclusions and the Lewy dendrites confirming the PD pathology (Figure 3C,D). Furthermore, immunohistochemistry revealed hyperphosphorylated protein tau within neurofibrillary tangles and dystrophic neurites in the hippocampus and striatum as seen in PART (Figure 3E). Focal areas within the dentate gyrus exhibited positive immunohistochemical staining of neuronal cytoplasm as well as some neuronal processes for TDP-43, with concurrent loss of expression of the protein in the neuronal nuclei (Figure 3F). Immunohistochemistry for Aβ yielded negative results. Molecular genetic analysis of the prion protein gene (*PRPN*) revealed no genetic mutations. The patient was homozygous for methionine 192.

## 3. Discussion

Rapidly progressive cognitive decline has diverse differential diagnoses that have to be considered and promptly acted on to manage possible reversible causes of cognitive deterioration. In this report, we present a patient with probable sCJD as the most likely diagnosis after detailed workup and exclusion of treatable causes. The clinical presentation of rapid cognitive decline, with additional ataxia, myoclonus, and finally akinetic mutism, was supported by characteristic findings on diagnostic tests such as cortical ribboning on MRI, positive 14-3-3 protein, highly elevated total tau in CSF together with periodic sharp wave EEG activity [16]. 

Previous studies of brain metabolic changes observed in sCJD patients, as captured by FDG PET, consistently demonstrated pronounced hypometabolism in various brain regions, including the caudate, thalami, middle and superior frontal gyri, parietal lobe, and posterior cingulum, along with relatively preserved metabolism in the medial temporal lobes [12,19,20]. While the distribution of hypometabolism in our patient generally corresponded to the expected pattern (although somewhat asymmetrically), the finding of relative hypermetabolism in the bilateral putamina was unexpected. Such a finding would typically be seen in PD [9], patients taking neuroleptics [21] (which he did not), or in some other rare circumstances which were unlikely in our case (e.g., isolated cases of autoimmune encephalitis, especially anti-LGI1 encephalitis [22,23], exposure to cocaine, amphetamines [21], interferon-α treatment [24], and some uncommon movement disorders of various etiology).

We additionally performed CJDRP expression analysis and due to hypermetabolism in the bilateral putamina also the PDRP expression analysis. Both network expressions were elevated compared to HS. While CJDRP expression exceeded 5.5 SD above HS, the expression of PDRP in our patient was relatively low, measuring 2.2 SD above the HS mean, which falls within the range typically seen in early PD. Previous studies have demonstrated a progression of PDRP expression scores throughout the course of the disease, with values around z = 2 in early stages, z = 3 in mid-stages, and over z = 4 in late stages [25]. Although our patient did not exhibit overt signs of parkinsonism, the early signs may have been masked by symptoms such as paratonia, ataxia, and overall slowness. The neuropathological examination, however, revealed the presence of Lewy bodies in the substantia nigra and locus ceruleus, consistent with Braak stage 3 [26]. It is worth noting that in Braak stage 3, many patients remain asymptomatic [27], yet they already exhibit PDRP expression [25].

Unfortunately, an assessment of nigrostriatal integrity in vivo through dopamine transporter scintigraphy/^18^F-fluorodopa PET or postmortem via immunohistochemical staining for tyrosine hydroxylase was not undertaken. Information on presynaptic dopaminergic integrity would have added valuable insights into the biological stage of PD in the presented patient as it corresponds to the disease duration and clinical manifestations, akin to the relationship observed with PDRP network expression.

Another intriguing pathological finding was the presence of hyperphosphorylated tau and TDP-43 pathology. To the best of our knowledge, this unique combination of four proteinopathies has not been documented previously. We hypothesize that a shared defect in protein homeostasis may have driven the pathological process, potentially involving excessive protein misfolding or impaired clearance of protein aggregates [28]. However, the presence of hyperphosphorylated tau and TDP-43 pathology was scant and probably did not have a significant impact on brain metabolism and networks.

This case provides a valuable insight into the intricate interplay within the neurodegenerative pathologies and their impact on metabolic brain characteristics. It highlights the value of metabolic brain imaging in diagnostic work-out of patients with neurodegenerative disorders in which multiple proteinopathies are often recognized, as it may point towards the underlying pathology in vivo.

## Data Availability

The data that support the findings of this study are available from the corresponding author, T.R., upon reasonable request.
